# Tendon-Specific Activation of Tenogenic Transcription Factors Enables Keeping Tenocytes’ Identity In Vitro

**DOI:** 10.3390/ijms232214078

**Published:** 2022-11-15

**Authors:** Rui Chen, Thomas Skutella

**Affiliations:** Group for Regeneration and Reprogramming, Institute for Anatomy and Cell Biology, Medical Faculty, Heidelberg University, Im Neuenheimer Feld 307, 69120 Heidelberg, Germany

**Keywords:** tendon tissue engineering, repair, animal model, decellularised extracellular matrix, mechanical stimulation

## Abstract

We generated a novel tetracycline-inducible transgenic mouse line with the tendon-specific expression of a series of tendon-critical transcription factors. Primary tenocytes derived from this mouse line consistently expressed green fluorescent protein reporter transcription factors in response to doxycycline. The tenocytes maintained their tendon cell properties for a longer time after the transient induction in the absence of growth factors and mechanical stress. Four key transcription factors for tendon development and the green fluorescent protein reporter were linked with different viral 2A self-cleaving peptides. They were expressed under the control of the tet-responsive element. In combination with the expression of BFP, which reports on the tendon-specific collagen I, and mScarlet, which reports on the tendon-specific transcription factor Scleraxis (Scx), we observed the more extended maintenance of the tendon cell identity of in vitro cultured tendon cells and Achilles tendon explants. This means that the Scleraxis bHLH transcription factor (Scx), mohawk homeobox (Mkx), early growth response 1 (Egr1) and early growth response 2 (Egr2) contributed to the maintenance of tenocytes’ identity in vitro, providing a new model for studying extracellular matrix alterations and identifying alternative biomaterials in vitro.

## 1. Introduction

The expression of many tendon-related genes changes with age, leading to tissue degeneration and subsequent injuries [1,2]. This partly explains older tendons’ limited endogenous repair capacity [3,4]. A few genes are clearly related to tendon development, but the genetic knockout of Scx, Mkx, Egr1 and Egr2 was shown to impair tendon function [5,6,7,8]. As a result of the expression of tendon-related genes, tenocytes maintain tendon function and homeostasis [9]. Whether the phenotype and function of tendon cells after an injury can be supported by regulating these key transcription factors during tendon development is the crucial question for the study of tendon reconstruction [6,10].

Scleraxis is one of the most critical transcription factors during tendon development [11]. After approximately 13.5 embryonic days (E), its expression becomes tendon-specific [12]. The level of expression decreases as the tendon matures and continues with ageing [11]. This explains, in some parts, why tendon repair becomes increasingly tricky with ageing [3]. Scx deficiency disrupts collagen synthesis and the organisation of the extracellular matrix [13,14]. This was shown to result in the impaired function of the force-transmitting tendons of mutant mice [15,16].

The knockout of Scx alone does not result in the complete loss of a tendon in neonatal mice [16]. This, on the one hand, implies that other transcription factors mediate tendon development. On the other hand, it suggests that the treatment of tendon injuries may be more effective through a strategy of multiple transcription factor combinations. Mohawk plays a crucial role in fibre alignment during tendon tissue development, and its deletion results in wavy tails in mutant mice [17]. Mkx overexpression prevents accidental ectopic ossification during tendon remodelling and enhances the reconstruction of the extracellular matrix [18,19].

In addition to Scx and Mkx, Egr1 and Egr2 are also involved in regulating tendon development through modifying the Scx and tendon extracellular matrix formation. Their knock-out results in the reduced expression of collagen type1 a1 (Col1a1) and reduced collagen fibrils in mutant mice [8]. Besides being involved in tendon development, Egr1 can also activate Col1a1 and collagen type2 a1 (Col2a1) via the bone morphogenic protein12 (BMP12)/Smad1/5/8 pathway after injury and regulate the transcriptional levels of Scx via transforming growth factor beta 2 (TGF-β2) signalling [20,21]. Its overexpression promotes mesenchymal stem cell (MSC) differentiation into the tendon lineage and ameliorates Achilles tendon injury in vivo [21].

**Figure 1:**
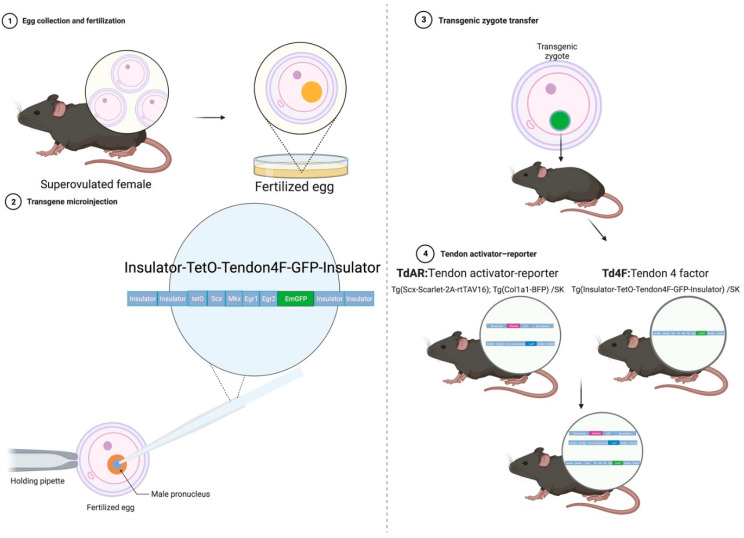
Scheme of TdAR-Td4F transgenic mouse generation. The constructed recombinant plasmids containing Insulator-TetO-Tendon4F-GFP-Insulator were linearised and purified. Then, Tg(Insulator-TetO-Tendon4F-GFP-Insulator)/SK for the tendon 4-factor transgenic mouse (Td4F) mouse line was obtained with a pronuclear microinjection [24]. Tg(Scx-Scarlet-2A-rtTAV16) Tg(Col1a1-BFP)/SK for the tendon activator–reporter (TdAR) transgenic mouse [22] was crossed with the Td4F mouse line. The above strategy obtained a novel inducible tendon-specific gene expression transgenic mouse strain: TdAR-Td4F mouse. (This figure was created using BioRender.com. The grey blocks represent genes, the green blocks represent green fluorescent protein reporter genes, the purple blocks represent mScarlet fluorescent protein reporter genes and the blue blocks represent blue fluorescent protein reporter genes.)

In summary, Egr1 and Egr2 better assist Scx and Mkx in supplementing tendon postinjury regeneration. Our generation of a tendon-specific dual fluorescent reporter transgenic mouse strain has made it possible to monitor cellular changes following tendon injury [22]. These tendon-specific reverse tetracycline transactivator (rtTA) transgenic mice contain Tet-On 3G (rtTAV16), which consists of three minimal VP16-activated structural domains. This system was reported to have a 100-fold improvement in doxycycline sensitivity compared to the original rtTA, while virtually eliminating the problem of the basal expression of its downstream genes in the absence of induction [23]. The rtTAV16, simultaneously driven by the Scleraxis promoter and reported on by the mScarlet red fluorescent protein, can induce the specific expression of manipulated genes in Scx-positive cells via the tetracycline response system (Tet-ON system) [22]. In contrast, the blue fluorescent protein reporter explicitly reports on the expression of tendon collagen I in the assessment of the regeneration of the tendon extracellular matrix (Figure 1).

## 2. Results

### 2.1. Generation of Four-Tendon Transcription Factor Transgenic Mice

The Insulator-TetO-Tendon4F-GFP-Insulator vector was linearised through restriction endonuclease enzyme digestion. Then, the purified fragments were microinjected into the pronuclei of 243 fertilised eggs. Then, the injected embryos were transferred into the oviducts of 10 pseudopregnant surrogate mothers. Thirty-five mice were obtained, and the birth rate of the transferred embryos was 14.4% (35/243). Two died before reaching one month of age, with a survival rate of born mice of 94.29% (33/35). The injected foreign gene was recombined into the genomic DNA of a portion of newborn mice [25]. To screen for newborn mice carrying the target gene, 33 founders were genotyped. A total of 10 founders had the target gene. Through the postinduction observation of progeny’s tendon cells, five of them were found to express the green fluorescent reporter in their line stably. One of the best founder strains was selected for the following characterisation experiments in vitro.

### 2.2. Tendon-Specific Expression of Four-Tendon Transcription Factor (Td4F)-EmGFP

#### 2.2.1. Expression of Scx-rtTA Is Tendon-Specific

In a previous work, we validated the TdAR transgenic mouse model [22]. Because both rtTA and mScarlet are driven by the Scx promoter, using mScarlet expression profiling, we verified that the expression of its upstream gene-rtTA was tendon-specific. In contrast, after exposure to doxycycline, the EmGFP expression was cloned downstream of the tetracycline response element (TRE: TetO sequences fused with a minimal human cytomegalovirus-derived promoter) and had the same distribution as the red fluorescence.

**Figure 2:**
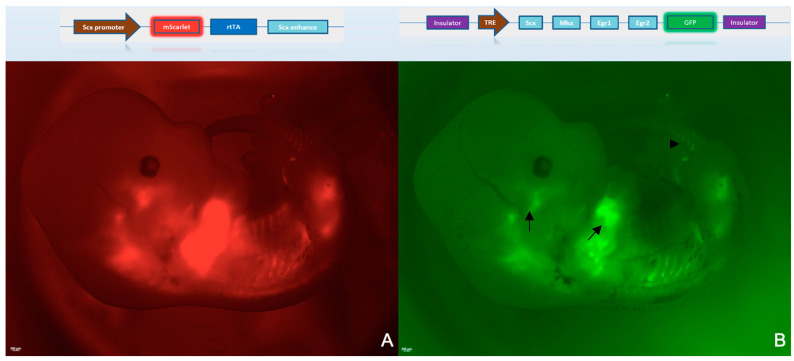
The Td4F-EmGFP expression pattern was consistent with the tendon region reported for Scx-mScarlet. To determine the region of induced expression of the Td4F transgene, we examined the green signal in whole-mount embryos using binocular fluorescence microscopy. The schematic above represents the Scx-rtTA-mScarlet and tetO-Td4F-EmGFP transgenes. (**A**) Scx-rtTA-mScarlet embryos at E12.5 under binocular fluorescence microscopy (×10 magnification). (**B**) tetO-Td4F-EmGFP embryo at E12.5 under binocular fluorescence microscopy (×10 magnification) (arrows show tendons around the developing maxillofacial and forelimbs; arrowheads show mScarlet expression in the tail).

At E12.5, the Scx+ tendon progenitor cells were concentrated at the tendon progenitor base in the limbs, ribs and head [26]. Through the use of binocular microscopy, it was observed that the mScarlet fluorescent protein was stably expressed in transgenic mouse embryos and entirely consistent with the region’s tendon-specific expression (Figure 2A). This implied that the reverse tetracycline-controlled transactivator-rtTA protein was located in these Scx+ tendon progenitors [22]. emGFP in the E12.5 transgenic mouse embryos faithfully recapitulated the tendon-specific nature of the induction system (Figure 2B). In TdAR-Td4F embryos of E12.5, the ligaments of the developing intracranial region outlined the ipsilateral masticatory and temporalis muscles [27]. EmGFP fluorescence in the limbs was brighter than in the connective tissue connecting the ribs and spinal regions, indicating the presence of a high enrichment in tendon progenitor cells, consistent with the higher Scx expression in the limb tendons than in the axial tendons [9].

By analysing the fluorescent protein expression in transgenic embryos containing Td4F- EmGFP transgenes, we validated the tendon-specific induction of four transcription factors, namely, Scx, Mkx, Egr1 and Egr2. This allowed for it to be possible to monitor the expression of combinations of the above transcription factors overexpressed in Scx+ cells during repair after tendon injury. Combined with the tendon reporter system, this allowed for the study of molecular therapy against tendon injury through the overexpression of the four transcription factors.

#### 2.2.2. EmGFP Is an Adequate Label for the Expression of Tendon Transcription Factors in Explants

In transgenic embryos of E12.5, the expression profile of Td4F-EmGFP overlapped with the tendon-specific Scx-mScarlet (Figure 2). To further analyse whether Td4F-EmGFP could be induced explicitly in the tendons of postnatal mice in Scx+ tendon cells, Achilles tendons from 6-week-old transgenic mice were collected and cultured as explants for the reporter gene analysis.

**Figure 3:**
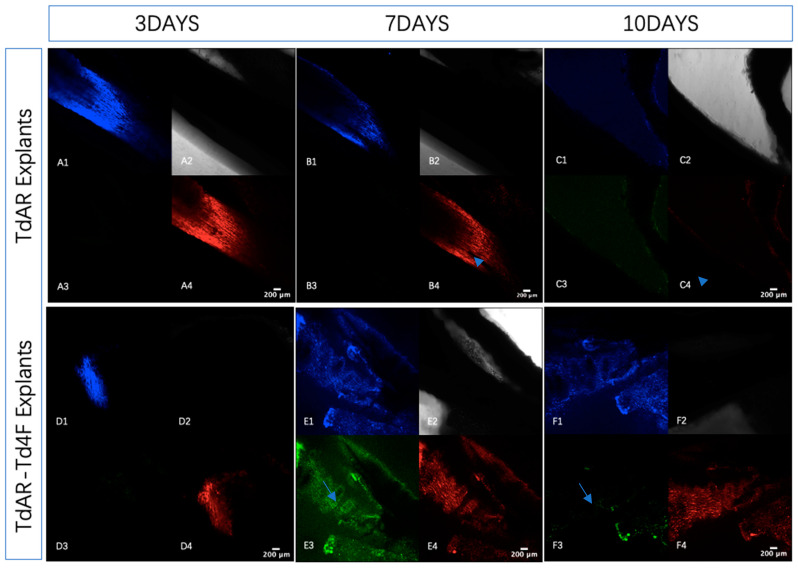
Transient Td4F overexpression in explant cultures prolonged the expression of Scx-Scarlet and Col1a1-BFP. For the in vitro analysis of tendon transcription factor expression in Achilles tendon cells, Achilles tendons from 6-week-old transgenic mice were collected. The expression of mScarlet and BFP was then progressively reduced in Achilles tendons deprived of mechanical traction during the culture of the Achilles tendon explants. The experiment was repeated three times independently using Achilles tendon explants from different donors. (**A**–**C**) show Achilles tendon explants from TdAR mice cultured for three, seven and ten days, respectively, under confocal microscopy (×10 magnification); **1**–**4** are views under confocal microscopy of Col1a1-BFP (**A1**–**F1**), brightfield (**A2**–**F2**), Td4F-EmGFP (**A3**–**F3**) and Scx-mScarlet (**A4**–**F4**) (×10 magnification). It could be observed that Scx-mScarlet and Col1a1-BFP gradually decreased in the absence of overexpression of Td4F. On day ten, the signal was almost completely unobservable, implying their loss of expression. (**D**–**F**) show Achilles tendon explants from TdAR-Td4F mice cultured for three, seven and ten days, respectively, under confocal microscopy (×10 magnification). (**E**) EmGFP was expressed in the explants after a transient induction following one day of exposure to doxycycline in culture, indicating that Td4F was overexpressed in the Scx+ cells of the explants. Expression of Scx-mScarlet and Col1a1-BFP was enhanced compared to (**D**). (**F**) Col1a1-BFP; Scx-mScarlet expression remained at high levels after Td4F-EmGFP expression was stopped (arrowheads indicate that Scx-mScarlet expression at the same position disappeared on day 10; arrows indicate that Td4F-EmGFP expression at the same position disappeared on day 10).

The outer sheath of the Achilles tendon essentially impairs the observation of fluorescent proteins using confocal microscopy. Therefore, the sheaths of the Achilles tendons were partially removed using a scalpel [28]. Then, the explants from TdAR and TdAR-Td4F mice were exposed to a culture medium containing doxycycline for 24 h to achieve transient Td4F-induced overexpression. Explants from TdAR-Td4F mice were observed to express Td4F-EmGFP the day after the induction, whereas no green fluorescent signal was detected in explants from TdAR mice (Figure 3B,E). This implied that the Td4F expression was induced and regulated by rtTA in the Scx-rtTA-mScarlet transgene.

The observation of mScarlet and BFP during the culture of Achilles tendon explants revealed that loss of mechanical traction resulted in a progressive decrease in the expression of Scx and Col1a1 in the explants (Figure 3A–C). This implied a progressive loss of identity and, consequently, the ability to produce type I collagen in tendon cells that were mechanically stimulated through the loss of traction due to the rupture of the Achilles tendons [29,30]. This was consistent with what was observed in vivo. Scar formation after tendon injury challenges the production of type I collagen, so the main component of the extracellular matrix at the site of injury becomes type III collagen [2,3,31,32]. Therefore, the ability to maintain the expression of Scx and Col1a1 in tendon cells after a tendon injury is critical for its reconstruction [2,3]. The expression of Scx-mScarlet and Col1a1-BFP was significantly enhanced after the transient overexpression of the combination of four transcription factors upregulated during tendon development (Figure 3D,E). The effect of the transient gene overexpression could be sustained at least until day 10, when the Td4F-EmGFP expression disappeared.

### 2.3. Td4F Contributes to the Maintenance of Tendon Cell Characteristics In Vitro

The complete removal of the tendon sheath is challenging and can easily damage the tissue structure. Therefore, we only partially removed the sheaths from the central location of the Achilles tendons. To observe the expression of Scx and Col1a1 in whole Achilles tendon explants, the explants were fixed and sectioned after 10 days in in vitro cultures. The fluorescent signals were observed in cross-sections of the entire explants under confocal microscopy.

**Figure 4:**
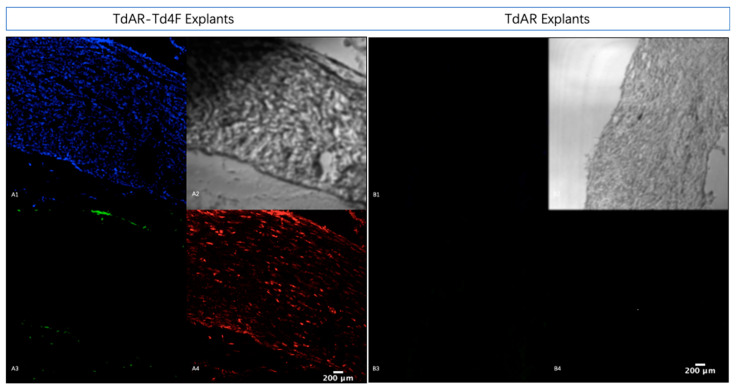
Td4F prolonged the expression of Scx and Col1a1 in explant cultures. For further observation of explants after ten days in in vitro cultures, explants were fixed and sectioned for observation under confocal microscopy. Compared to explants from TdAR mice, Achilles tendon explants from TdAR-Td4F mice were found to remain stably expressed in mScarlet and BFP after Td4F-EmGFP overexpression had ceased. (**A**) Frozen sections of Achilles tendon explants from TdAR-Td4F mice cultured for ten days in vitro under confocal microscopy (×10 magnification). (**B**) Frozen sections of Achilles tendon explants from TdA mice cultured in vitro for ten days under confocal microscopy (×10 magnification); **1**–**4** show observations of Col1a1-BFP (**A1**,**B1**), brightfield (**A2**,**B2**), Td4F-EmGFP (**A3**,**B3**) and Scx-mScarlet (**A4**,**B4**) under confocal microscopy, respectively (×10 magnification).

After 24 h of doxycycline induction, high levels of mScarlet and BFP expression could be sustained until the Td4F-EmGFP expression ceased (Figure 4A,B). This implied that the transient overexpression of Td4F as a strategy could have a long-term effect after the overexpression ends. This shows potential for the transient induction of long-term benefits for gene therapies based on this strategy [33].

### 2.4. Four Tendon Transcription Factors Enhance the Expression of Tendon-Specific Genes In Vitro

**Figure 5:**
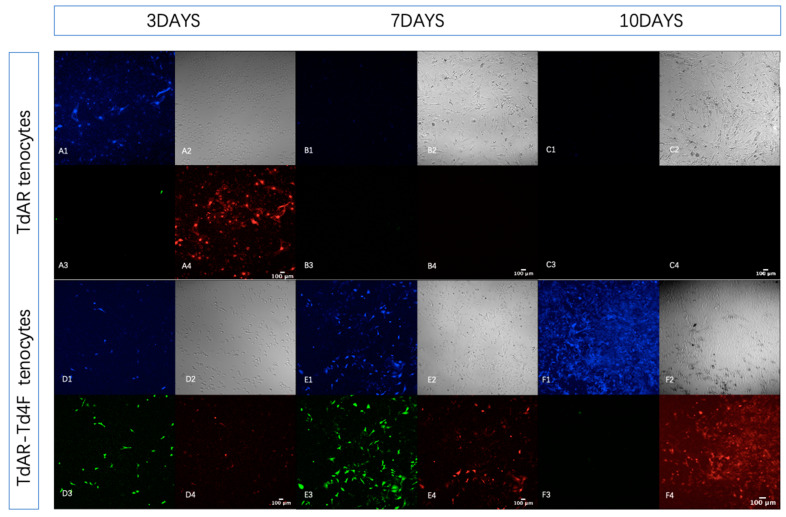
Td4F increased Scx-mScarlet and Col1a1-BFP expression in Scx+ primary Achilles tendon cells in vitro. To confirm, at the cellular level, the increased expression of Scx-mScarlet and Col1a1-BFP in Scx+ primary Achilles tendon cells after induction of Td4F-EmGFP overexpression, tendon cells were extracted from the Achilles tendons of TdAR mice and TdAR-Td4F mice, respectively, for 24 h of induction. The experiment was repeated three times independently, using cells from different donors. (**A**–**C**) show primary Achilles tenocytes from TdAR mice cultured under confocal microscopy for three, seven and ten days (×10 magnification); **1**–**4** show Col1a1-BFP (**A1**–**F1**), brightfield (**A2**–**F2**), Td4F-EmGFP (**A3**–**F3**) and Scx-mScarlet (**A4**–**F4**) (×10 magnification) in confocal microscope view. It can be seen that in the absence of overexpression of Td4F, Scx-mScarlet and Col1a1-BFP gradually decreased. At ten days, the signal was almost completely unobservable, implying that these genes lost expression. (**D**–**F**) show views of tendon cells from TdAR-Td4F mice cultured for three, seven and ten days, respectively, under confocal microscopy (×10 magnification). (**D**) One day after exposure to doxycycline culture, EmGFP expression began in tendon cells from TdAR-Td4F mice, indicating that Td4F was significantly overexpressed in Scx+ cells from TdAR-Td4F mice. (**E**) Td4F overexpression resulted in enhanced expression of Scx-mScarlet and Col1a1-BFP compared to three days. (**F**) Expression of Col1a1-BFP and Scx-mScarlet remained at high levels after Td4F-EmGFP expression was stopped.

To confirm, at the cellular level, the increased expression of Scx-mScarlet and Col1a1-BFP in Scx+ primary Achilles tendon cells following the induction of Td4F-EmGFP overexpression, tendon cells were extracted from the Achilles tendons of 6-week-old TdAR mice and TdAR-Td4F mice, respectively, and subjected to a 24 h induction. col1a1-BFP and Scx- mScarlet were upregulated after the Td4F overexpression, reflecting that Td4F increased the expression of Scx-mScarlet and Col1a1-BFP in Scx+ primary Achilles tendon cells in vitro (Figure 5D–F). Additionally, after the cessation of Td4F-EmGFP expression, the expression of Col1a1-BFP and Scx-mScarlet remained at high levels, which validated the results in explants (Figure 4D–F). In contrast, in Scx+ primary Achilles tendon cells from TdAR mice that did not overexpress Td4F, Scx-mScarlet and Col1a1-BFP gradually reduced, and more rapidly too compared to in explants. At seven days, the signal almost wholly disappeared, meaning that the expression of the fluorescent protein reporter gene ceased (Figure 5A–C).

**Figure 6:**
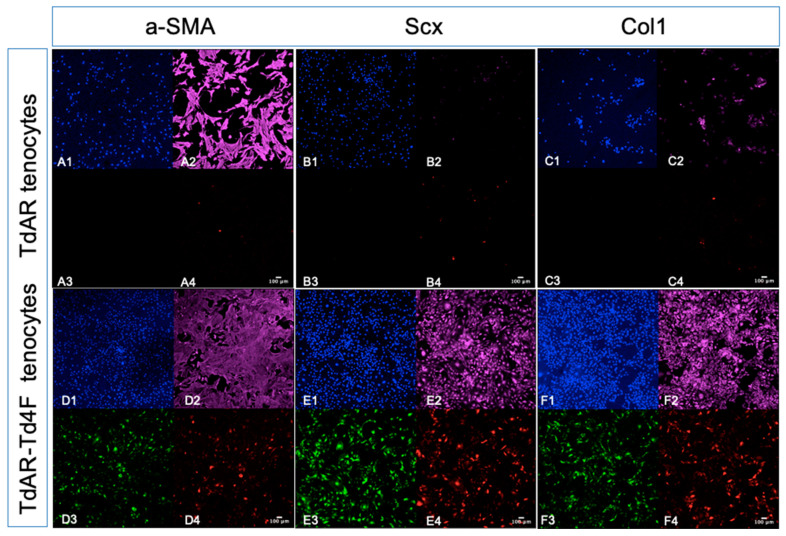
Overexpression of Td4F in tenocytes upregulated Scx and Col1a1 while reducing a-SMA expression. To further confirm the expression of Scx and Col1a1 and the fibrosis marker a-SMA in Scx+ primary Achilles tenocytes after induction of Td4F-EmGFP overexpression, we applied immunohistochemical staining (IHC) to tenocytes cultured for seven days using antibodies against Scx, Col1a1 and a-SMA. (**A**–**C**) show the results of staining for a-SMA, Scx and Col1a1 in primary Achilles tenocytes from TdAR mice under confocal microscopy (×10 magnification); **1**–**4** show the confocal DAPI staining (**A1**–**F1**), immunofluorescence staining (**A2**–**F2**), Td4F-EmGFP (**A3**–**F3**) and Scx-mScarlet (**A4**–**F4**) (×10 magnification). Focused microscope views. (**D**–**F**) show the results of staining for a-SMA, Scx and Col1a1 in primary Achilles tenocytes from TdAR-Td4F mice under confocal microscopy (×10 magnification). It can be seen that the expression of Scx-mScarlet was significantly higher in the case of the overexpression of Td4F. (**A2**,**D2**) Td4F overexpression reduced a-SMA expression in tenocytes, implying that Td4F has an antifibrotic potential. (**B2**,**E2**) Td4F overexpression increased Scx expression in tenocytes, implying that Td4F expression maintained tenocyte identity. (**C2**,**F2**) Td4F overexpression increased Col1a1 expression in tendon cells, suggesting that Td4F expression could promote tendon recovery after injury.

To further study the expression of Scx and Col1a1 and the fibrosis marker a-SMA in Scx+ primary Achilles tendon cells after the induction of Td4F-EmGFP overexpression, we applied immunohistochemical staining (IHC) to the cultured Achilles tendon cells for seven days using antibodies against Scx, Col1a1 and a-SMA. Primary Achilles tendon cells from TdAR mice and TdAR-Td4F mice were transiently induced for 24 h (medium containing 1 µg/mL doxycycline), and primary Achilles tendon cells from TdAR-Td4F mice showed an increased expression of mScarlet at seven days (Figure 6D4–F4). The overexpression of Td4F was found to reduce a-SMA expression in tendon cells through cellular immunofluorescence (Figure 6A2,D2), implying that Td4F has an antifibrotic potential. Td4F overexpression also increased the expression of Scx (Figure 6B2,E2) and Col1a1 (Figure 6C2,F2) in tendon cells, implying that Td4F expression maintained tendon cell-specific genes and has the potential to promote tendon recovery after injury.

## 3. Discussion

We generated and characterised a new tetracycline-inducible transgenic mouse strain that could tendon-specifically overexpress transcription factors to investigate the role of combinations of highly expressed transcription factors on tendon cell identity and their functional maintenance during tendon development. In combination with tendon-specific double-reporter transgenic mice, this allowed for the possibility to study the alterations in the lineage and function of tendon explants and tenocytes through Td4F overexpression-based gene therapy.

Scx+ tendon stem/progenitor cells (TSPCs) are major cells that have been shown to aid in repairing injured tendons of neonatal mice [34], which partly explains why tendon repair is far better in juveniles than in old age. The Tppp3+Pdgfra+ fibrous-lipogenic progenitor cells that are a potential source of tendon stem cells were located in the surrounding tissue of the tendons (sheaths). After a tendon injury, they moved to the site of injury, where the TSPCs converted to Scx+ and were responsible for repairing the tendon injury. At the same time, a subpopulation of Scx- cells formed scars [35]. Therefore, we aimed to maintain tenocyte properties and promote the conversion of tendon repair cells to Scx+ TSPCs by overexpressing Td4F in the absence of beneficial growth factors [36,37] and mechanical stimulation [36,38] (the case after tendon injury).

Our results showed that Td4F upregulated Scx and Col1a1 while reducing a-SMA expression in tendon cells. This implied that Td4F was antifibrotic and promoted the repair of the tendon extracellular matrix. td4F significantly increased the number of Scx-mScarlet-expressing cells in explants, suggesting that Td4F could potentially boost the conversion of fibrous-lipogenic progenitor cells entering the injury site to Scx+ tendon stem/progenitor cells to Scx+ tendon stem/progenitor cells. This means that Td4F could potentially be used in gene therapy for tendon injuries, as the Td4F transient overexpression strategy was free from the limitations of mechanical stimulation and growth factors. It did not require long-term induction, and, therefore, Td4F overexpression-engineered cells also have the potential to be transformed into a cell therapy for the treatment of tendon injuries [37,39].

## 4. Materials and Methods

### 4.1. Cloning of the tetO-Td4F-EmGFP Transgenic Constructs

The Scleraxis bHLH transcription factor (Scx), mohawk homeobox (Mkx), early growth response 1 (Egr1) and early growth response 2 (Egr2) and tetracycline operator (tetO) DNA separate from pCMV6-mMkx-DDK-tag (Origene: 225168), pCMV6-mScx-DDK-tag (Origene: 202202), pCMV6-mEgr1-DDK-tag (Origene: 227136), pcDNA 3.1-mouse Egr2 (pcDNA 3.1(-) mouse Egr2 were a gift from Peter Johnson, Addgene plasmid #107,997;http://n2t.net/addgene:107997, accessed on 21 March 2019; RRID:Addgene_107997) and TetO-FUW-OSKM (TetO-FUW-OSKM was a gift from Rudolf Jaenisch, Addgene plasmid #20,321; http://n2t.net/addgene:20321, accessed on 15 February 2019; RRID: Addgene_20321) were amplified in Q5 High-Fidelity 2X Master Mix (New England Biolabs, Ipswich, MA, USA), then inserted into a vector with the insulator by NEBuilder HiFi DNA Assembly Master Mix. Using the same strategy, an EmGFP fragment was inserted to obtain a recombinant plasmid containing insulators, the tetracycline response element, Td4F and EmGFP.

After being linearised with EcoRV and SalI, the TetO-4TF-EmGFP transgenic fragment was isolated for the prenuclear microinjection.

### 4.2. Achilles Tendon Explants and Tenocytes Culture In Vitro

Achilles tendon explants and tenocytes were isolated according to protocol separately from Costa-Almeida et al. [40] and Seluanov et al. [41]. The tissue fragments were transferred into a 2 mL DMEM medium. Then, 200 µL of Liberase Blendzyme III (Roch) stock solution (10 mg/mL) was added to the medium and incubated overnight in a cell incubator. The cells and tissue debris were centrifuged at 1200 rpm for 5 min to precipitate the cells and tissue debris. The supernatant was discarded and the mixture was transferred, including the tissue fragments, to a 2 mL culture medium (DMEM, supplemented with 20% FBS, penicillin/streptomycin, Doxycycline and Amphotericin B). Achilles tendon explants and tenocytes were incubated in a humidified 5% CO_2_ incubator at 37 °C. The medium was changed daily to fresh medium until all tissue debris was washed away. The tenocytes at passage 2–3 were used for all experiments.

Twelve Achilles tendons were collected from three TdAR mice and three TdAR-Td4F transgenic mice at six weeks of age. Six Achilles tendons from the left leg were used to extract the tendon cells. A total of 6 right Achilles tendon tissues was used for the Achilles tendon explant cultures. The above experiments were repeated separately for three rounds.

### 4.3. Immunohistochemistry and Immunohistochemistry

The Achilles tendon explants were washed in PBS and fixed overnight at 4 °C in freshly prepared 4% PFA solution. The samples were washed thoroughly three times in PBS at 4 °C for one hour each. Whole-mount embryos were photographed with a binocular microscope. Immersion in 10%, 20% and 30% sucrose solutions was conducted for the pre-embedding protection of the cryosections. Then, they were embedded in OCT and placed on dry ice to solidify for long-term storage in a −80 °C refrigerator. The tissue blocks were left overnight in the −20 °C refrigerator for sectioning the next day. The temperature of the Cryostat microtome (Leica CM3050S, Wetzlar, Germany) was adjusted and the tissue block trimmed. Serial sections of explants had a 10 µm thickness. The cut tissue pieces were mounted on SuperFrost slides and stored in the dark at −20 °C. The photographs were captured using a confocal microscope (Carl Zeiss LSM900, Jena, Germany) and processed with the Fiji software Mac OS X (US National Institutes of Health).

The slides were washed three times with PBS to remove impurities. The cells were fixed with 4% PFA for 30 min at room temperature. the cells were washed three times with PBS for 10 min each. The cells were permeabilised in PBS containing 0.1% Triton X-100 for 15 min to permeabilise the cell membrane. Endogenous cellular antigens were blocked with 5% BSA for 1 h at room temperature. Then, they were incubated with a primary antibody and incubated overnight at 4 °C. The following primary antibodies were used: anti-Scx (1:500; ab58655), anti-aSMA (1:500; ab124964) and antitype I collagen (1:500; ab21286). The cells were washed three times with PBS for 10 min each. The cells were, subsequently, incubated with secondary antibodies Alexa Fluor 647 IgG H&L (1:1000; ab150083) for 1 h at room temperature. The cells were washed three times with PBS for 10 min each time. DAPI and stain were added for 10 min for nuclear staining. Cells were washed three times with PBS for 10 min each. After flow-through rinsing, coverslip slides with Mowiol 488 (Roche, #0718) were used.

## Data Availability

The datasets generated and analysed during the current study are available from the corresponding authors upon reasonable request.
